# Far-Infrared Ameliorates Pb-Induced Renal Toxicity via Voltage-Gated Calcium Channel-Mediated Calcium Influx

**DOI:** 10.3390/ijms242115828

**Published:** 2023-10-31

**Authors:** Chin-Meng Ko, Chee-Kin Then, Yu-Ming Kuo, Yen-Kuang Lin, Shing-Chuan Shen

**Affiliations:** 1 Graduate Institute of Medical Sciences, College of Medicine, Taipei Medical University, Taipei 11031, Taiwan; d119104004@tmu.edu.tw (C.-M.K.); m120107020@tmu.edu.tw (Y.-M.K.); 2 Department of Radiation Oncology, Shuang Ho Hospital, Taipei Medical University, New Taipei City 23561, Taiwan; b101100141@tmu.edu.tw; 3 Graduate Institute of Athletics and Coaching Science, National Taiwan Sport University, Taoyuan 33301, Taiwan; 4 Department of Dermatology, School of Medicine, Taipei Medical University, Taipei 11031, Taiwan; 5 International Master and Ph.D. Program in Medicine, College of Medicine, Taipei Medical University, Taipei 11031, Taiwan

**Keywords:** far-infrared (FIR), cellular protection, lead (Pb) toxicity, nephrotoxicity, voltage-gated calcium channel (VGCC), calcium influx

## Abstract

Far-infrared (FIR), characterized by its specific electromagnetic wavelengths, has emerged as an adjunctive therapeutic strategy for various diseases, particularly in ameliorating manifestations associated with renal disorders. Although FIR was confirmed to possess antioxidative and anti-inflammatory attributes, the intricate cellular mechanisms through which FIR mitigates lead (Pb)-induced nephrotoxicity remain enigmatic. In this study, we investigated the effects of FIR on Pb-induced renal damage using in vitro and in vivo approaches. NRK52E rat renal cells exposed to Pb were subsequently treated with ceramic-generated FIR within the 9~14 μm range. Inductively coupled plasma mass spectrometry (ICP-MS) enabled quantitative Pb concentration assessment, while proteomic profiling unraveled intricate cellular responses. In vivo investigations used Wistar rats chronically exposed to lead acetate (PbAc) at 6 g/L in their drinking water for 15 weeks, with or without a concurrent FIR intervention. Our findings showed that FIR upregulated the voltage-gated calcium channel, voltage-dependent L type, alpha 1D subunit (CaV1.3), and myristoylated alanine-rich C kinase substrate (MARCKS) (*p* < 0.05), resulting in increased calcium influx (*p* < 0.01), the promotion of mitochondrial activity, and heightened ATP production. Furthermore, the FIR intervention effectively suppressed ROS production, concurrently mitigating Pb-induced cellular death. Notably, rats subjected to FIR exhibited significantly reduced blood Pb levels (30 vs. 71 μg/mL; *p* < 0.01), attenuated Pb-induced glomerulosclerosis, and enhanced Pb excretion compared to the controls. Our findings suggest that FIR has the capacity to counteract Pb-induced nephrotoxicity by modulating calcium influx and optimizing mitochondrial function. Overall, our data support FIR as a novel therapeutic avenue for Pb toxicity in the kidneys.

## 1. Introduction

Chronic kidney disease (CKD) is a growing public health problem worldwide, characterized by impaired renal function and increased mortality rates [1]. Notably, lead (Pb) exposure was identified as a risk factor associated with renal dysfunction [2,3]. Pb-induced renal damage impairs renal function through various mechanisms, including increased oxidative stress caused by the generation of reactive oxygen species (ROS), the inhibition of antioxidation enzymes, severe inflammation, mitochondrial dysfunction, and ATP depletion, ultimately leading to renal cell death [4,5,6,7]. Additionally, Pb disrupts calcium homeostasis, further contributing to Pb-induced cytotoxicity [8,9]. In pharmacokinetic studies, it was observed that changes in calcium concentration have a significant impact on the absorption of Pb [10], indicating an inverse relationship between the concentrations of calcium and Pb in the body. The competitive interaction between calcium and Pb might be due to their similar ionic characteristics, common utilization of ion channels, and competition for binding sites [11]. Those properties collectively contribute to the reduction in intracellular Pb concentration when calcium is present in higher concentrations. A previous study showed that Pb can impair the function of calcium channels by changing their structure by binding to calcium-binding sites or undergoing oxidative–reductive modifications, resulting in neuronal cell death [12]. Although details of the mechanism in the kidneys are still unclear, it is evident that Pb-induced nephrotoxic responses may be closely related to calcium disturbances [10].

Far-infrared (FIR) radiation is a novel intervention for CKD [13,14]. FIR exposure demonstrated beneficial effects such as enhancing vascular conditions, including increasing arterial blood flow and peripheral blood circulation [15], reducing blood pressure, promoting capillary dilation [16], promoting tissue healing [17,18,19], and protecting cells from damage [20,21,22]. FIR radiation is an invisible electromagnetic wave within the range of 3 to 1000 μm [23] that exerts its effects through thermal and non-thermal mechanisms. Despite the known protective effects of FIR radiation in complications of CKD [24], the underlying molecular mechanisms in renal tissues remain unclear.

Intriguingly, previous studies suggested that electromagnetic radiation can modulate calcium concentrations by influencing the opening of calcium channels and promoting calcium influx [25,26,27]. For example, Li et al., reported that terahertz waves enhanced the permeability of voltage-gated calcium channels (VGCCs), thereby regulating calcium flux across cells [28]. These studies revealed that electromagnetic radiation can influence calcium concentrations. As a type of electromagnetic radiation, FIR also regulates intracellular calcium levels. FIR was shown to increase nitric oxide (NO) production by activating a signaling axis involving intracellular calcium mobilization, Ca^2+^/calmodulin-dependent protein kinase II (CaMKII) activation, and endothelial NO synthase (eNOS)-Ser1179 phosphorylation in bovine aortic endothelial cells [29]. In addition, Hsu et al., found that FIR radiation promoted beta-cell insulin secretion in diabetic mice, which was related to activation of the CaV1.2 VGCC [22].

Given the association between Pb-induced toxic responses and disruptions in calcium homeostasis, as well as the ability of electromagnetic radiation to modulate calcium concentrations, it was hypothesized that FIR radiation may have the potential to ameliorate Pb-induced cytotoxicity in the kidneys. Therefore, this study aimed to investigate the potential pathway through which FIR radiation exerts protective effects against Pb-induced nephrotoxicity. Our findings revealed that FIR radiation increased the expressions of VGCCs, leading to calcium influx and intracellular Pb reductions. This provides evidence that FIR radiation ameliorated Pb-induced nephrotoxicity by regulating calcium influx via the activation of VGCCs.

## 2. Results

### 2.1. FIR Exposure Reduced Pb-Induced Cytotoxicity in Renal Proximal Tubular Cells

**Figure 1:**
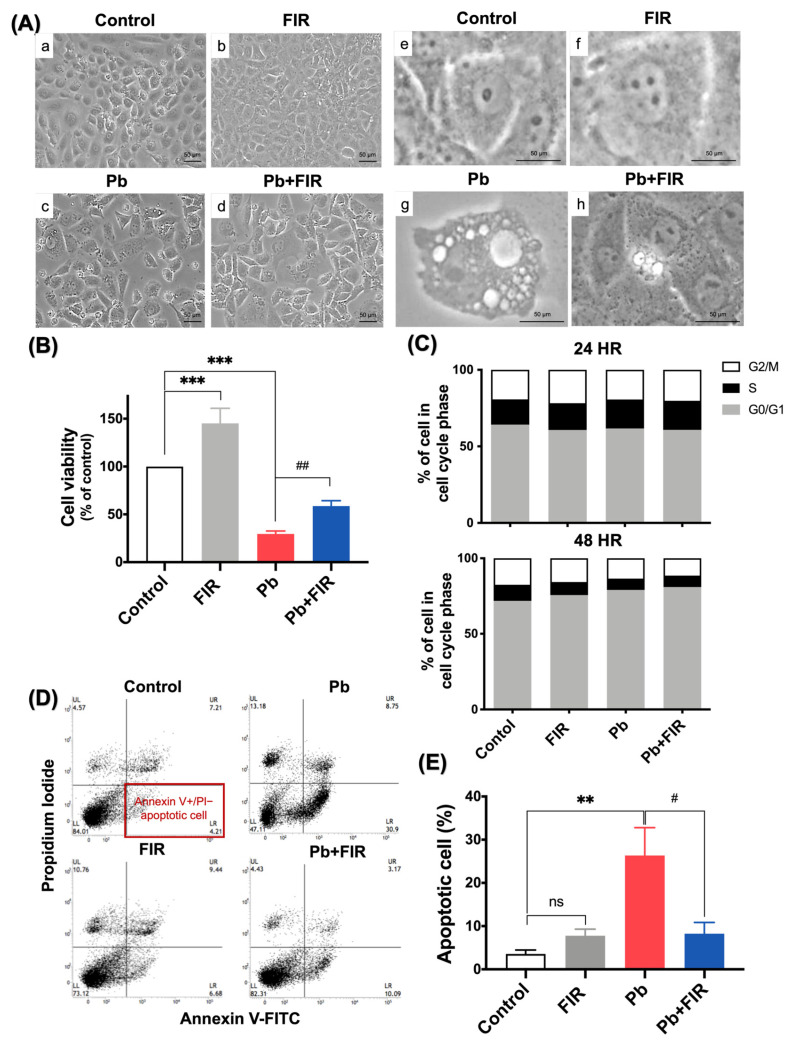
Far-infrared (FIR) exposure reduced lead (Pb)-induced cell death. (**A**) Microscopic observations of NRK52E cells with or without 72 h of FIR exposure, showing Pb-induced alterations in cell morphologies. Panels (**a**–**d**): magnification ×40; panels (**e**–**h**): magnification ×100. (**B**) Cell viability was assessed via an MTT assay. The viability of the control group was normalized to 100%. (**C**) Analysis of cell cycle alterations via flow cytometry, monitoring DNA contents of cells after Pb treatment with or without 24 and 48 h of FIR exposure. Results show no significant changes, and cells at each cell cycle phase were quantitatively assessed by counting the number of cells. (**D**,**E**) Apoptotic cells were assessed by AV/PI staining, and the percentage of apoptotic cells was measured via flow cytometry. Data were collected from three independent experiments and are expressed as the mean ± SD. Statistical significance: ** *p* < 0.01; *** *p* < 0.001 compared to the control; ^#^ *p* < 0.05; ^##^ *p* < 0.01 compared to the Pb group; ns, not significant.

To determine whether FIR protects renal proximal tubular cells from Pb-induced toxicity, we treated NRK52E cells pretreated with PbAc by exposing them to FIR. Morphological changes in the cells were observed, and cell viability was assessed using an MTT assay. As shown in Figure 1A, the Pb group exhibited observable apoptotic bodies and cellular vacuolization (in panel g), whereas in the Pb + FIR group, these phenomena exhibited improvements. These observations suggest that FIR may have protective effects against Pb-induced toxicity. In line with these results, an MTT assay (Figure 1B) demonstrated that 200 μM PbAc significantly suppressed NRK52E cell viability (*p* < 0.001). However, suppression was prevented in the Pb + FIR group (*p* < 0.01; Figure 1B). These results further support the protective role of FIR against Pb-induced toxicity. Additionally, FIR exposure alone significantly promoted the proliferation of NRK52E cells (Figure 1B, FIR group). Interestingly, increased cell proliferation in the FIR group was independent of changes in the cell cycle (Figure 1C). Furthermore, an Annexin V/propidium iodide (PI) assay revealed that the Pb group exhibited a 26% increase in cell apoptosis. However, when comparing the Pb + FIR group to the Pb group, we found that the percentage of apoptotic cells was reduced to 8% (Figure 1D,E). Our results indicate that FIR had a protective effect against Pb-induced cell death, further supporting its role in mitigating Pb-induced toxicity.

### 2.2. FIR Exposure Decreased Intracellular Pb Concentrations

**Figure 2:**
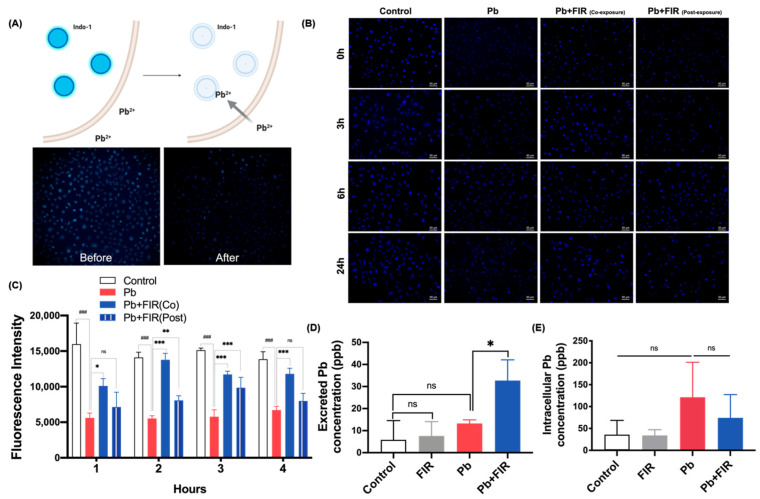
Far-infrared (FIR) exposure reduced intracellular lead (Pb) concentrations by promoting Pb outflow. (**A**) Indo-1, a fluorescent dye, was used as a tool for Pb detection in cells. Pretreatment of cells with Indo-1 allowed the fluorescence to be quenched upon binding with Pb ions. (**B**) Fluorescence microscopic images illustrating intracellular Pb detection using Indo-1 staining of NRK52E cells after high-dose PbAc (400 μM) treatment, with or without FIR exposure. Control: untreated cells; Pb: 400 μM PbAc treatment for 2 h; Pb + FIR (co-exposure): 400 μM PbAc treatment for 2 h with simultaneous FIR exposure; Pb + FIR (post-exposure): FIR exposure following 400 μM PbAc treatment for 2 h (scale bar = 50 μm). (**C**) fluorescence intensity measurements. (**D**,**E**) Determination of excretion and uptake of Pb concentrations in NRK52E cells using ICP-MS. Mean ± SD of three independent experiments. Statistical significance: * *p* < 0.05; ** *p* < 0.01; *** *p* < 0.001 compared to the Pb group; ^###^ *p* < 0.001 compared to the control group; ns, not significant.

To explore the protective effects of FIR against Pb toxicity, we examined whether the decrease in Pb-induced toxicity was related to a reduction in intracellular [Pb^2+^] levels. To investigate this, we utilized the fluorescent dye Indo-1 to assess Pb accumulation in cells. The fluorescence of Indo-1 was quenched upon binding to cytosolic Pb within cells, as demonstrated in Figure 2A. Before Pb exposure, cells were pretreated with Indo-1 (left panel), and the degree of fluorescence quenching was measured as an indicator of Pb uptake (right panel). Upon addition of the PbAc solution, the fluorescence intensity gradually decreased within 2 min and rapidly disappeared between 4 and 8 min (Figures S1, S2 and Video S1), suggesting the rapid entry of Pb into cells. The fluorescence intensity of the Pb group was significantly quenched within 3 h, and the Pb group exhibited the highest intracellular Pb concentrations among all the groups (Figure 2B, lane 2). In contrast, FIR exposure strikingly restored fluorescence responses in the Pb + FIR (co-exposure) and Pb + FIR (post-exposure) groups (Figure 2B, lanes 3 and 4), indicating that FIR exposure effectively reduced intracellular Pb concentrations. Notably, the fluorescence intensity of the Pb + FIR (co-exposure) group was higher than that of the Pb + FIR (post-exposure) group, implying that FIR exposure might not only promote Pb excretion but also interfere with Pb uptake (Figure 2B, lanes 3 and 4). In the quantitative results (Figure 2C), a similar trend was observed. To assess the contribution of FIR exposure to promoting Pb excretion from cells, we measured intracellular and extracellular Pb concentrations. In addition, a baseline measurement (after washing residual Pb) was established to confirm that the observed increase in Pb excretion was a direct result of FIR exposure, and not influenced by residual Pb interference in the extracellular medium. Pb concentrations in the culture medium were determined at 0 (baseline) and 72 h. The values obtained after subtraction represent the concentration of Pb excretion. The Pb group without FIR exposure exhibited a Pb excretion concentration of 10.8 ppb, indicating a natural ability of Pb-treated cells to excrete a small amount of Pb. However, with FIR exposure, Pb excretion significantly increased to 32.7 ppb (*p* < 0.01; Figure 2D) in the Pb + FIR group. Moreover, we analyzed intracellular Pb concentrations, which were lower in the Pb + FIR group (100 ppb) compared to the Pb group (121 ppb) (Figure 2E). These results suggest that FIR exposure may reduce intracellular Pb levels by promoting Pb excretion from Pb-treated cells, potentially contributing to its protective effect against Pb-induced toxicity. Our findings indicate that the protective effect of FIR against Pb toxicity might be linked to a reduction in intracellular [Pb^2+^] levels.

### 2.3. Mitigation of ROS Production, ATP Depletion, and Mitochondrial Dysfunction following FIR Exposure in the Presence of Pb Treatment

**Figure 3:**
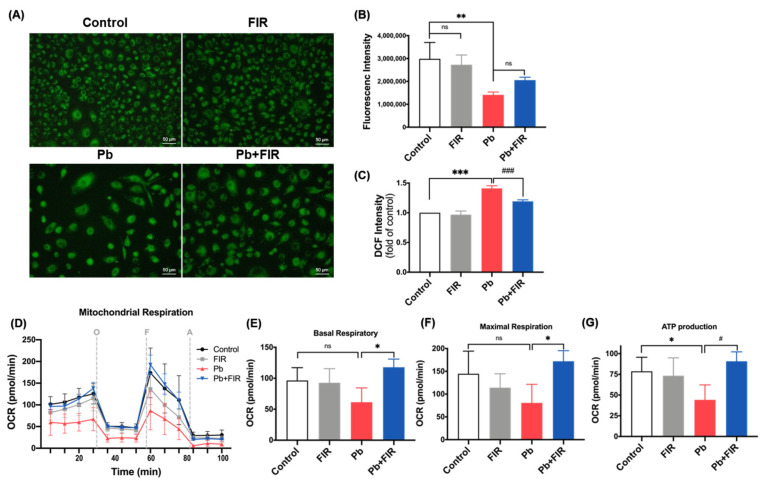
Mitigation of reactive oxygen species (ROS) production, ATP depletion, and mitochondrial dysfunction by far-infrared (FIR) exposure in the presence of lead (Pb) treatment. (**A**) Fluorescence images of mitochondria in cultured NRK52E cells were obtained using MitoTracker dye. Magnification: ×40 in panels. (**B**) Quantitative data of MitoTracker fluorescence intensity. (**C**) Quantification of ROS production at 48 h following FIR exposure, assessed using DCF fluorescence intensity measured via flow cytometry. Data are presented as fold changes compared to the untreated control. Mean ± SD of three independent experiments. (**D**) Measurement of mitochondrial respiration levels, indicated by the oxygen consumption rate (OCR), in all groups with or without 50 μM PbAc treatment and FIR exposure for 72 h under basal conditions, or following the addition of the ATP synthase inhibitor oligomycin (O; 1 μM), the uncoupler FCCP (F; 2 μM), and the electron transport inhibitor antimycin A (A; 0.5 μM). (**E**) Quantification of the basal respiration rate and (**F**) quantification of the maximal respiratory capacity, determined by normalizing OCR levels. (**G**) Assessment of ATP production. Statistical significance: ^#,^* *p* < 0.05, ** *p* < 0.01, ^###,^*** *p* < 0.001; ns, not significant.

To demonstrate whether intracellular [Pb^2+^] reduction is sufficient to protect cells or mitigate cellular damage. ROS production and mitochondrial dysfunction are well-established indicators of Pb toxicity [30]. Furthermore, previous studies suggest that FIR can enhance mitochondrial function [21]. Thus, we hypothesized that FIR exposure may improve cell survival and mitigate cytotoxicity by enhancing or preserving mitochondrial activity. To assess the mitochondrion status in living cells, we utilized MitoTracker fluorescence and conducted microscopic observations. As shown in Figure 3A, Pb treatment resulted in a decrease in the number of mitochondria, indicating mitochondrial dysfunction. Interestingly, FIR exposure had no significant effect on mitochondrion quantity. Our quantitative results further confirmed that the Pb group exhibited a significant decrease in mitochondrial numbers, which was not observed in the Pb + FIR group (Figure 3B). We also examined whether FIR could inhibit Pb-mediated ROS production. DCF-DA analysis showed that Pb treatment significantly increased ROS production in the Pb group at 48 h compared to the control group (*p* < 0.001; Figure 3C), whereas the Pb + FIR group showed a significant decrease in ROS production compared to the Pb group (*p* < 0.001, Figure 3C). To further investigate the impact of FIR on mitochondrial function following Pb treatment, we measured oxygen consumption rates (OCRs). The Pb group (red line) displayed a reduced OCR, suggesting a decline in the overall metabolic rate, compared to the control group (black line; Figure 3D). However, this difference was not observed in the Pb + FIR group (blue line). Our data demonstrated a decrease in the basal respiratory rate, maximal respiratory rate, and ATP production in the Pb group. However, FIR exposure after Pb treatment not only increased the mitochondrial respiratory rate and ATP production but also restored the respiratory capacity in cells (Figure 3E–G), suggesting that FIR exposure enhanced the mitochondrial respiratory capacity in cells exposed to Pb toxicity. Our findings indicate that FIR exposure effectively reduced intracellular [Pb^2+^] levels, enhanced the mitochondrial respiratory capacity, and robustly inhibited ROS production. These effects collectively contributed to promoting cell viability and reducing Pb-induced cellular damage through FIR exposure.

### 2.4. Investigating Mechanism of FIR-Induced Intracellular Pb Efflux Based on Increased Expressions of VGCCs and Subsequent Calcium Influx

**Figure 4:**
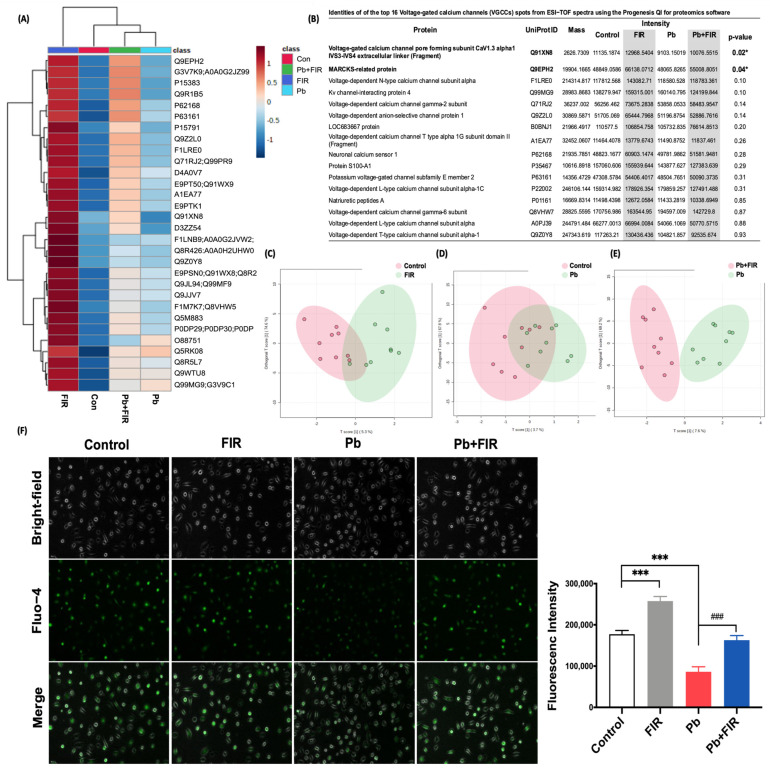
Proteomics analysis of voltage-gated calcium channels (VGCCs) in NRK52E cells treated with far-infrared (FIR) and its effect on calcium influx. (**A**) Heatmap depicting differential expressions of identified proteins (*m*/*z*) between the control and FIR-treated groups and the Pb− and Pb+ FIR-treated groups. (**B**) A table presenting the identified VGCC proteins. Statistical analysis was conducted using Progenesis QI for the proteomics analysis and MetaboAnalyst. (Three independent replicate measurements were performed for each group.) (**C**–**E**) Orthogonal PLS-DA plot analysis via LC QTOF MS, classifying groups into control vs. FIR, control vs. Pb, and Pb vs. Pb + FIR. (**F**) Intracellular calcium levels were evaluated in cells after high-dose PbAc (400 μM) treatment, with or without FIR exposure for 3 h. This assessment was performed using Fluo-4 staining, followed by quantification through fluorescence microscopy and intensity measurements (scale bar = 100 μm). Mean ± SD of three independent experiments. Statistical significance: *** *p* < 0.001 compared to the control group; * *p* < 0.05 and ^###^ *p* < 0.001 compared to the Pb group.

To investigate the mechanism underlying the protective effects of FIR in reducing Pb levels and its potential impacts on cellular protection, we conducted a comprehensive proteomic analysis to identify proteins associated with FIR-induced Pb excretion. Utilizing quadrupole time of flight mass spectrometry (QTOF-MS), a powerful analytical tool for protein characterization and enhanced precision, we aimed to unravel protein alterations implicated in this process. The results demonstrated a significant increase in proteins associated with VGCCs upon FIR treatment (Figure 4A, lanes 1 and 3). Among the proteins identified, two noteworthy candidates emerged: the CaV1.3 voltage-gated calcium channel pore-forming subunit (Q91XN8) and the myristoylated alanine-rich C kinase substrate (MARCKS)-related protein (Q9EPH2) (*p* < 0.05; Figure 4B). Notably, the Pb + FIR group exhibited the highest mean expression levels of these proteins compared to the Pb group. Further proteomic analysis using the Progenesis QI for proteomics software (version 2.4.6911.27652) confirmed fold changes exceeding 1.2, with confidence scores of 30.18 for CaV1.3 and 59.1 for the MARCKS-related protein. To visualize differences between samples within each group, we performed a partial least squares discriminant analysis (PLS-DA). This analysis indicated distinct protein expression profiles associated with FIR exposure and Pb treatment (Figure 4C–E). Our findings suggest that FIR exposure induced the upregulation of VGCCs, particularly CaV1.3 and MARCKS-related proteins. However, it is yet to be determined whether the observed increase in CaV1.3 and MARCKS-related protein expression directly influenced calcium levels or Pb alterations. To elucidate the potential pathway by which FIR regulates ion mobilization, we investigated alterations in intracellular calcium concentrations using Fluo-4 dye. The quantitative data (Figure 4F) revealed strongly lower Fluo-4 intensity in the Pb group compared to the control group (*p* < 0.001), indicating a decrease in intracellular calcium concentrations in Pb-treated cells. Conversely, the Pb + FIR group exhibited significantly increased Fluo-4 intensity (*p* < 0.001), suggesting elevated cytosolic calcium concentrations following FIR exposure. Overall, our findings suggest that FIR exposure promoted the upregulation of VGCCs, particularly CaV1.3 and MARCKS-related proteins, which likely play roles in mediating calcium influx. These findings enhanced our understanding of how FIR-induced changes contribute to the reduction in intracellular Pb levels and provided insights into the potential mechanisms underlying the protective effects of FIR against Pb-induced cellular damage.

### 2.5. FIR Ameliorated Pb-Induced Nephrotoxicity through Decreased Blood Pb Levels and Enhanced Pb Excretion in Wistar Rats

**Figure 5:**
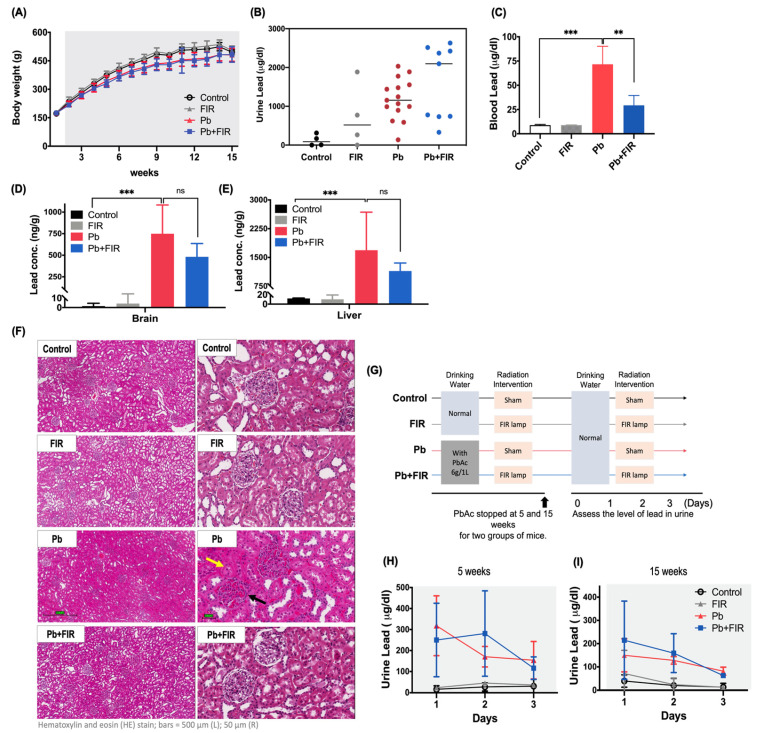
Far-infrared (FIR) ameliorated lead (Pb)-induced nephrotoxicity in Wistar rats. Wistar rats were administered PbAc in their drinking water for a duration of 15 weeks. (**A**) Body weight curve of all experimental groups. (**B**) Measurement of urine Pb concentrations at 9 weeks. (**C**) Measurement of blood Pb concentrations at 15 weeks. (**D**) Pb concentration analysis in brain and (**E**) liver tissues, assessed through ICP-MS detection, at 15 weeks. (**F**) H&E-stained renal sections. The control and FIR groups exhibited normal structures of proximal tubules and glomeruli. Proximal tubular cell injury is denoted by a yellow arrow, and glomerular injury is indicated by a black arrow (scale bar = 50 μm). Wistar rats were exposed to drinking water containing PbAc (6 g/L) for 5 and 15 weeks, followed by discontinuation of Pb water exposure. (**G**) Experimental groups: control (no treatment); FIR (40 min of daily FIR exposure); Pb (no FIR exposure); Pb + FIR (40 min of daily FIR exposure). Daily urine samples were collected for Pb excretion analysis. (**H**) Urine Pb concentration at 5 weeks (short-term treatment). (**I**) Urine Pb concentration at 15 weeks (long-term treatment). Mean ± SD of three independent experiments. Statistical significance: *** (*p* < 0.001) when compared to the control group, ** (*p* < 0.01) when compared to the Pb + FIR group for blood, brain, and liver Pb concentrations; ns, not significant. Sample sizes: *n* = 5 for the control and FIR groups; *n* = 22 for the Pb group; *n* = 14 for the Pb + FIR group.

Finally, we validated whether FIR ameliorated Pb-induced nephrotoxicity in an animal model. Wistar rats, known for their high tolerance to Pb toxicity and suitability as in vivo models for Pb toxicity research [31], were employed for this investigation. As shown in Figure 5A, there were no significant differences in weight among the Pb, Pb + FIR, FIR, and control groups during the experimental period. Based on in vitro findings, we further examined whether FIR exposure could reduce Pb accumulation in this rat model. Urinary Pb levels were measured to assess Pb excretion. The data (Figure 5B) showed that the Pb + FIR group had higher urinary Pb levels compared to the Pb group at 9 weeks, indicating that long-term exposure to Pb combined with FIR may accelerate Pb excretion through urine.

Subsequently, after sacrificing the rats, blood samples were collected, and Pb concentrations were measured. The data (Figure 5C) showed that FIR exposure alone did not affect blood Pb concentrations, but Pb treatment significantly increased blood Pb concentrations to 71 μg/dL. FIR exposure significantly reduced blood Pb concentrations in the Pb + FIR group (71 μg/dL for Pb vs. 30 μg/dL for Pb + FIR; *p* < 0.01), indicating that FIR exposure mitigated the Pb-induced elevation in blood Pb concentrations. Moreover, Pb accumulation in the brain and liver tissues was analyzed. The Pb + FIR group had lower Pb accumulation levels in the brain and liver tissues compared to the Pb group (Figure 5D,E). Histopathological analyses (Figure 5F) further supported that FIR exposure mitigated Pb-induced kidney damage. We observed indications of proximal tubular cell and glomerular injury in the H&E-stained sections. Additionally, we investigated whether FIR could promote Pb excretion through urine. The experimental design (Figure 5G) involved short- and long-term Pb exposure, followed by the discontinuation of Pb intake and initiation of FIR treatment at 5 and 15 weeks, respectively. Daily urine samples were collected for Pb concentration measurements. The results revealed that the peak of Pb excretion in urine occurred on the second day after discontinuing Pb intake and receiving FIR treatment. We observed a non-significant trend of increased Pb excretion in the Pb + FIR group at 5 and 15 weeks (Figure 5H,I). In our study, we observed that FIR exposure mitigates Pb-induced renal injury in animal models, in line with the results observed in in vitro experiments.

## 3. Discussion

FIR is a type of low-risk, side-effect-free electromagnetic radiation for managing diseases [13,32,33,34,35,36,37]. Abundant clinical and basic research supports FIR’s protective properties, indicating its potential to alleviate diseases by inhibiting inflammation and promoting mitochondrial activities [21,22,38]. We established an FIR electromagnetic radiation-treated cell model to examine its protective effects against Pb-induced nephrotoxicity. We found that FIR exposure upregulated the expressions of CaV1.3 VGCC and MARCKS. This upregulation potentially led to increased calcium influx and interference with Pb concentration in cells, as evidenced by the decreased Pb accumulation and increased Pb excretion in Pb-treated cells. Consequently, FIR exposure had the ability to reduce Pb accumulation in cells, alleviate Pb-induced toxic responses, and mitigate renal damage. Taken together, our findings provide valuable insights into the protective effects of FIR and its potential to reduce Pb toxicity.

Electromagnetic radiation can regulate cellular processes by affecting the membrane potential and ion channels, leading to changes in intracellular ion concentrations [39]. Electromagnetic radiation may directly cause variations in the membrane potential, subsequently triggering the opening of calcium channels and allowing an influx of calcium ions. Interfering with the potential difference across cell membranes can increase calcium channel permeability, allowing more calcium ions to enter cells [25,26,27]. Alternatively, electromagnetic radiation might induce the opening of calcium channels, leading to an influx of calcium ions into cells. This influx of calcium ions could then impact changes in the membrane potential [25,26]. The relationship between electromagnetic radiation and the membrane potential is complex and multifaceted. Although it is evident that electromagnetic radiation can affect ion channels and intracellular calcium concentrations, further research is needed to establish the precise cause-and-effect relationship regarding changes in the membrane potential.

FIR is a form of electromagnetic radiation that may share similar regulatory pathways, and a growing body of evidence has indicated that low-frequency, low-energy electromagnetic radiation can increase the penetration and movement of calcium ions across cell membranes through calcium channels, leading to increased intracellular calcium concentrations [28,40,41]. Although limited research has been conducted on the cellular mechanisms of FIR, similar findings were reported that stated that FIR may involve the regulation of calcium channels, leading to calcium influx [22,29,42]. Herein, we analyzed the calcium channel expressions of NRK52E cell lines under FIR exposure through QTOF. We observed that the expressions of two VGCCs were upregulated in NRK52E cells exposed to FIR (Figure 4B). Our results revealed the novel finding that FIR promotes activity of the CaV1.3 and MARCKS calcium channels. These are closely associated with calcium influx. Notably, CaV1.3 was reported to be expressed in the kidneys, where it increases calcium influx into renal cells [43].

Calcium plays a vital role in cell survival and apoptosis-regulatory pathways [44]. In our study, we observed that FIR protected NRK52E cells against Pb-induced cell death by increasing intracellular calcium concentrations (Figure 4). Despite the complexity of calcium signaling, our findings consistently demonstrate FIR’s protective effects in both cellular and animal models. Similar results were reported in other studies [22,42,45], suggesting the potential relevance of calcium concentrations and regulatory pathways in different cell types. This evidence emphasizes the importance of calcium in cellular responses to FIR and supports FIR’s protective effects. Understanding the underlying mechanisms involved in FIR-mediated calcium regulation could pave the way for further research.

Pb interference with calcium metabolism and balance in cells is well documented in several studies [11,46,47]. This interference affects vital biological processes, including signal transduction, protein synthesis, and cell growth, ultimately leading to tissue and organ damage and dysfunction [11,12]. Furthermore, various studies have reported on the relationship between Pb and calcium ions within cells [48,49]. Based on these findings, we propose the hypothesis of a competitive relationship between calcium and Pb. Our research demonstrated that FIR exposure promotes the influx of calcium into cells while concurrently reducing intracellular Pb levels (Figure 2). As a result, this intervention effectively mitigates Pb-induced renal toxicity (Figure 1). Pharmacokinetic modeling studies revealed that calcium concentrations exert a significant effect on Pb absorption. High calcium concentrations reduce Pb absorption and transfer, whereas low calcium concentrations increase Pb absorption and transfer [10]. However, it is important to acknowledge that current investigations into Pb absorption and transfer primarily rely on modeling, lacking clear experimental evidence to explain dynamic changes within cells. Although we observed an increase in calcium influx due to FIR exposure, we were unable to provide direct experimental evidence to explain the subsequent decrease in Pb intracellular levels. Similar to previous research, the challenge remains in obtaining conclusive empirical evidence regarding the dynamic changes involved in Pb absorption and transfer within cells.

Previous studies have explored the relationship between calcium channels and the entry of Pb ions [50,51,52]. While previous research indicated that Pb can share calcium ion channels for cellular entry [53,54], our research offers a new perspective. By activating specific VGCCs through FIR, we observed increased intracellular calcium and a simultaneous decrease in cellular Pb concentrations. This intriguing result suggests that FIR might shield cells from Pb toxicity, possibly through mechanisms involving calcium-Pb equilibrium and competitive interactions at the channel level. The balance between calcium and Pb ions plays a critical role. The activation of VGCCs elevates intracellular calcium, driving calcium from regions of high concentration to regions of low concentration. Concurrently, lower extracellular Pb levels may encourage the exiting of Pb from cells. Additionally, the activation of calcium channels might lead to competitive interactions between calcium and Pb, impacting their respective entry rates [10,48]. These interconnected factors likely contribute to the observed decrease in intracellular Pb concentrations. Our results substantiate this proposition. The activation of specific VGCCs via FIR effectively reduced cellular Pb contents, reinforcing our argument. Remarkably, this trend was consistent across both cellular and animal experiments, providing validation for our position.

**Figure 6:**
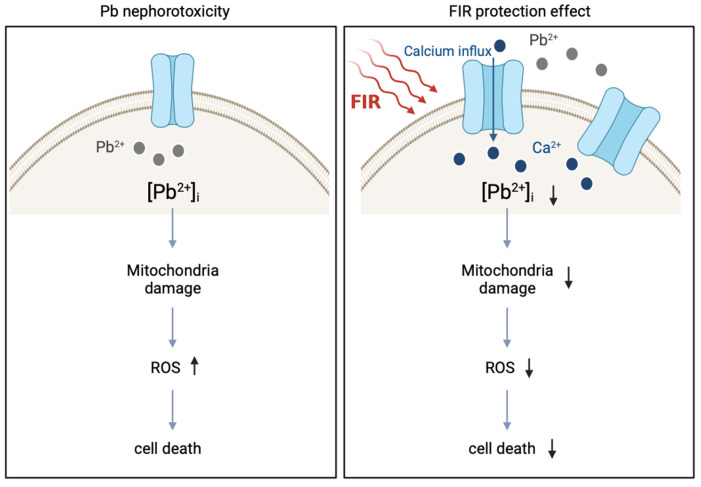
Schematic overview of the protective effects of far-infrared (FIR) in lead (Pb)-treated NRK52E cells. FIR exposure activates voltage-gated calcium channel (VGCC) proteins, thereby regulating the influx of calcium into cells. The increase in intracellular calcium concentrations may facilitate the excretion of intracellular Pb, resulting in the attenuation of Pb-induced reactive oxygen species (ROS) production and mitochondrial dysfunction. Ultimately, these protective mechanisms contribute to the restoration of cell viability.

To the best of our knowledge, this is the first study investigating the potential effects of FIR on Pb excretion, Pb toxicity amelioration, and renal cell protection via VGCC activation. Our findings suggest that FIR achieves these effects by upregulating VGCCs, leading to increased ion movements, and potentially reducing intracellular Pb levels via calcium influx. Moreover, FIR appears to enhance mitochondrial activity, resulting in increased ATP production, which may counteract Pb-induced toxicity (Figure 6). In the past, FIR radiation was believed to have resonant interactions with water molecules, promoting beneficial effects in organisms’ growth and development. Our study proposes that FIR may exert a protective effect by upregulating VGCC expressions in cell membranes. While we have made progress in understanding the interactions between FIR and cellular processes involving calcium in Pb toxicity, it remains unclear whether FIR also influences other critical ions and their downstream reactions. Surprisingly, our investigation revealed unexpected effects on other ion channels, suggesting that FIR might not only regulate calcium ion movement but also impact other ions through specific voltage-gated channel proteins. Further in-depth research is needed to fully comprehend the physiological mechanisms underlying FIR’s actions.

Our study has several limitations that should be addressed. First, the mechanism by which FIR electromagnetic radiation activate VGCCs remains elusive, necessitating further investigations to establish a causal relationship. Additionally, we acknowledge certain gaps in our understanding of the cellular mechanisms underlying the effects of calcium concentrations and Pb toxicity. We did not determine the correlation between the dynamic changes in calcium influx and Pb reductions after FIR exposure. Additionally, in our animal study, it is important to note that H&E staining has limitations in definitively assessing glomerulosclerosis. As a consideration for future research, the use of more specific staining methods, such as PAS or trichrome staining, would be valuable for a more comprehensive evaluation. Despite these limitations, we observed a clear trend of decreased Pb concentrations during FIR exposure, which may indicate a possible link to VGCC activation and calcium influx. This observed trend is consistent with the amelioration of Pb-induced nephrotoxicity during FIR exposure. Currently, obtaining direct evidence to establish clear causal relationships for all of the examined outcomes has proven challenging. As a result, we have discussed and provided reasonable explanations for our findings. Given these limitations and shortcomings, conducting experiments to elucidate the regulatory mechanism underlying the interactions between FIR electromagnetic radiation and calcium ion channels, and their effects on ion concentrations and toxicity, is essential for future research. Our study indicates that FIR may represent a novel therapeutic approach for mitigating Pb toxicity in renal cells.

## 4. Materials and Methods

### 4.1. FIR Exposure

FIR radiation was produced using an FIR ceramic semiconductor (YIN FU RUI DE Co., Ltd., Tainan, Taiwan) to generate 9~ and 14 μm wavelengths (Figure S3A) at a power density of 13.14 W/cm^2^. Pure FIR ceramic without a power supply was spread under cell culture plates and covered with aluminum foil in a 37 °C incubator with a 5% CO_2_ atmosphere (Figure S3B,C). Simultaneously, a control group covered with aluminum foil was set up under the same conditions. In the animal study, a CH-8222 emitter (FIR lamp) was set up 25 cm above the bottom of the cages, and Wistar rats were exposed to FIR radiation for 40 min/day for 15 weeks.

### 4.2. Cell Culture and Pb Treatment

Proximal tubule epithelial cells (NRK52E) were obtained from ATCC (Manassas, VA, USA) and cultured in Dulbecco’s modified Eagle medium (DMEM) supplemented with 5% bovine serum (BS) in a CO_2_ incubator at 37 °C. Cells were grown to 80~90% confluence and were seeded in 24-well plates for a 3-(4,5-dimethylthiazol-2-Yl)-2,5-diphenyltetrazolium bromide (MTT) assay and Seahorse test; in 3.5 cm dishes for fluorescence staining; and in 6 cm dishes for inductively coupled plasma mass spectroscopy (ICP-MS) and Western blot analyses. In all experiments, cells at passages 25~35 were used. NRK52E cells were pretreated with 200 or 400 μM lead acetate (PbAc) for 30 min, and then, washed with phosphate-buffered saline (PBS). Cells treated with PbAc were exposed to the FIR ceramic as described above.

### 4.3. Cell Viability

The cellular survival rate was determined using an MTT assay. NRK52E cells were seeded at 2.5 × 10^4^ cells/well in 24-well plates. To determine the effects of the pure FIR ceramic, cells were divided into four groups: the control group was maintained under normal conditions; the FIR group consisted of cells treated with FIR ceramic only; the Pb group consisted of cells pretreated with PbAc; and the Pb + FIR group consisted of cells pretreated with PbAc, and then, exposed to the FIR ceramic. Cells were incubated at 37 °C for 72 h. MTT was prepared at a concentration of 500 μg/mL in DMSO, and 20 μL of the MTT solution was added to each well. Plates were then incubated in a 37 °C incubator for 3 h. After incubation, 200 μL of isopropanol was added to dissolve the formazan crystals. The resulting solution was transferred to a 96-well enzyme-linked immunosorbent assay (ELISA) plate (Corning Incorporated, Corning, NY, USA), and the absorbance was measured at 595 nm. The optical density (OD) of the control group was normalized to 100%.

### 4.4. Cell Apoptosis Analysis

Cells (5 × 10^5^) in 3.5 cm dishes were washed with PBS and stained with 5 μL of Annexin V-FITC and 10 μL of propidium iodide (PI) in 200 μL of binding buffer for 10 min at room temperature in the dark. Apoptotic cells were detected using an Annexin V/PI assay kit (ALX-850-250-KI02, Enzo Life Sciences, Plymouth Meeting, PA, USA) following the manufacturer’s instructions. Fluorescence intensity was measured using a flow cytometer (FACScan, Becton Dickinson, Sunnyvale, CA, USA).

### 4.5. Analysis of Mitochondrial Morphology

Cells were seeded in 3.5 cm dishes, stained with 1 mM Mitotracker Green (Invitrogen, Carlsbad, CA, USA), and incubated at 37 °C for 40 min, following the manufacturer’s instructions. Cell mitochondrial images were captured using a Zeiss Axio Observer Z1 fluorescence microscope (Zeiss, Taipei, Taiwan) and analyzed using ZEN 2.3 PvCam image software (Taiwan Instrument, Taipei, Taiwan).

### 4.6. Seahorse XF24 Assay

NRK52E cells (0.65 × 10^4^ cells/well) were seeded in a Seahorse XF cell culture plate for 72 h. To determine the mitochondrial function, the oxygen consumption rate (OCR) was detected using a Seahorse Biosciences XF24 Analyzer (Agilent Technologies, Santa Clara, CA, USA). According to the manufacturer’s protocol, the cell culture medium was replaced with assay medium (DMEM without sodium bicarbonate) and maintained at 37 °C for 1 h before completing probe calibration. The basal respiratory rate was measured using the Seahorse Biosciences XF24 Analyzer. Up to three compounds were added to the assay cartridge via automated injection during the assay: 1 μM oligomycin, 2 μM FCCP, and 0.5 μM rotenone. The added compounds caused no cell death during the mitochondrial energy analysis.

### 4.7. Intracellular ROS Production

Cells (10^4^) were incubated for 48 h with or without FIR exposure, washed twice with PBS, and stained with 10 μM dihydrofluorescein diacetate (DCFH-DA, Invitrogen). Levels of ROS were measured as fluorescence intensities via FACScan flow cytometry using a flow cytometer (FACScan, Becton Dickinson) at an excitation wavelength of 488 nm and an emission wavelength of 530 nm.

### 4.8. Intracellular Calcium ([Ca^2+^]_i_) and Lead ([Pb^2+^]_i_) Measurements

To measure intracellular calcium concentrations, cells were incubated with 2 μM Fluo-4-AM (Molecular Probes, Eugene, OR, USA) at 37 °C for 45 min. Subsequently, cells were examined using a Zeiss Axio Observer Z1 fluorescence microscope equipped with ZEN PvCam image software to capture real-time [Ca^2+^]_i_ images. To measure intracellular lead concentrations, the fluorescent probe Indo-1 (Molecular Probes, Invitrogen) was utilized to detect the cytosolic entry of Pb^2+^ ions. Cells were stained with 1 μM Indo-1-AM for 45 min at 37 °C, following the protocol described in Kerper et al., and other relevant publications [55,56].

### 4.9. Analysis of Pb Concentrations via ICP-MS

ICP-MS is a highly sensitive assay used to detect specific metal ions. All solutions utilized in this experiment were prepared using ultrapure water with a resistivity of 18.2 MΩ·cm. Samples were digested using 69–70% nitric acid (HNO_3_, J.T. Baker, Phillipsburg, NJ, USA) at a temperature of 95 °C for a duration of 2 h.

The analysis sequence consisted of a standard addition curve created with an increasing order of concentrations, followed by blanks and samples. All samples were diluted 50 times with deionized water to a final volume of 10 mL. Samples were filtered through polytetrafluoroethylene membrane filters prior to analysis using an Agilent 7800 ICP-MS system (Agilent Technologies). To ensure accurate results, the ICP-MS introduction system was rinsed with 2% HNO_3_ between each sample, and a standard curve was analyzed to verify the stability of the instrument.

### 4.10. Proteomic Analysis

A specific liquid chromatographic quadrupole–time-of-flight mass spectrometric (LC-QTOF-MS) analysis was used for the protein analysis in this study. Sample preparation was performed as described by Reni Ajoy et al. [57]. The progenesis QI system (Waters, Milford, MA, USA) was used for proteomic analysis. Peptides were recognized via ion accounting identification from the Uniport rat database, which includes VGCC-related proteins. Search parameters were adjusted, including static modification of cysteine (carbamidomethylation) and variable modifications of methionine (oxidation), asparagine, and glutamine (deamidation). The protein peptide false-discovery rate was set to <4%. Data were analyzed using MetaboAnalyst 5.0 to generate heatmaps and partial least squares discriminant analysis (PLS-DA) diagrams for comparisons and statistical analyses. Differentially expressed peptides were identified as 1.5-fold changes in intensity between two groups of samples.

### 4.11. Animals

The animal study protocol was confirmed and approved by the Institutional Animal Care and Use Committee or Panel (IACUC/IACUP) at Taipei Medical University, approval No. LAC2023-0067 (6 April 2023). Male Wistar rats (4 weeks old, weighing 101~125 g) obtained from BioLasco (Taipei, Taiwan) were used in this study. Animals were randomly divided into four groups. Rats in the control and FIR groups were fed daily with normal water and treated with sham or FIR exposure for 40 min. Pb and Pb + FIR group rats were daily treated with lead acetate (PbAc, 6 g/L) in drinking water and with sham or FIR exposure for 40 min/day at a distance of 25 cm for 15 weeks. During the entire experimental period, rats were maintained on a 12:12 light–dark cycle at a constant temperature of 25 °C. All rats were allowed ad libitum access to food and water.

### 4.12. Sample Collection In Vivo

At the time of blood collection, a heavy metal-free anticoagulant was added to the sampling tubes. All blood samples were stored at 4 °C prior to analysis of Pb concentrations. Fresh urine samples were collected in heavy metal-free tubes and stored at −20 °C for the Pb concentration assay. Samples of liver, brain, and kidney tissues were collected for histopathological analyses or freeze-dried for ICP-MS analyses.

### 4.13. Statistical Analysis

All data are presented as the mean ± standard deviation (SD) of three independent experiments. A two-way analysis of variance (ANOVA) with Bonferroni post hoc analysis was applied to assess differences among experimental conditions, with the significance level set at *p* < 0.05.

## 5. Conclusions

In conclusion, far-infrared (FIR) possesses the potential to enhance Pb excretion, ameliorate Pb toxicity, and provide protection to renal cells. These processes may involve reducing intracellular Pb concentrations via calcium influx, alongside enhancing mitochondrial ATP production to counteract Pb-induced toxicity. Our study results suggest that FIR might assume a role in upregulating ion channel expression, thereby bestowing a protective effect. Furthermore, our findings indicate that VGCC expressions, specifically CaV1.3 and MARCKS, were positively correlated with calcium influx. The progress we have made in comprehending the interplay between FIR and cellular processes, especially its exploitation of calcium ions to mitigate Pb toxicity, is noteworthy. However, a comprehensive grasp of the underlying mechanisms of FIR demands further exhaustive investigations in subsequent endeavors.

## Data Availability

All data generated or analyzed during this study are included in the published article.

## Supplementary Materials

The following supporting information can be downloaded at: https://www.mdpi.com/article/10.3390/ijms242115828/s1.
