# Supplementary material for: Far-Infrared Ameliorates Pb-Induced Renal Toxicity via Voltage-Gated Calcium Channel-Mediated Calcium Influx
Source: Int J Mol Sci. 2023 Oct 31;24(21):15828. doi: 10.3390/ijms242115828 (PMC10649088; doi:10.3390/ijms242115828)
Supplement: Supplementary file 1 [file ijms-24-15828-s001.zip › Supplementary Data.pdf]

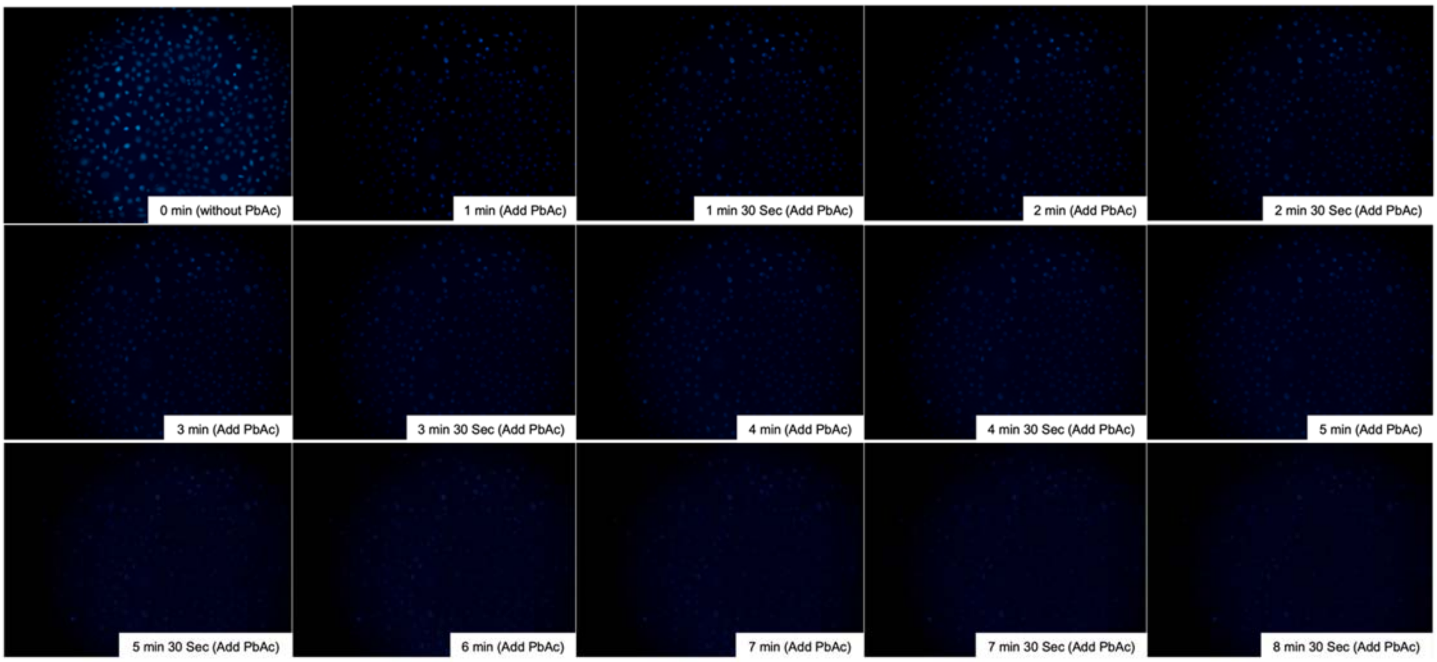

Figure S1. Image: Time-Dependent Fluorescence Intensity of Indo-1 Changes upon PbAc Solution Addition. Scale bar: 50 µm.

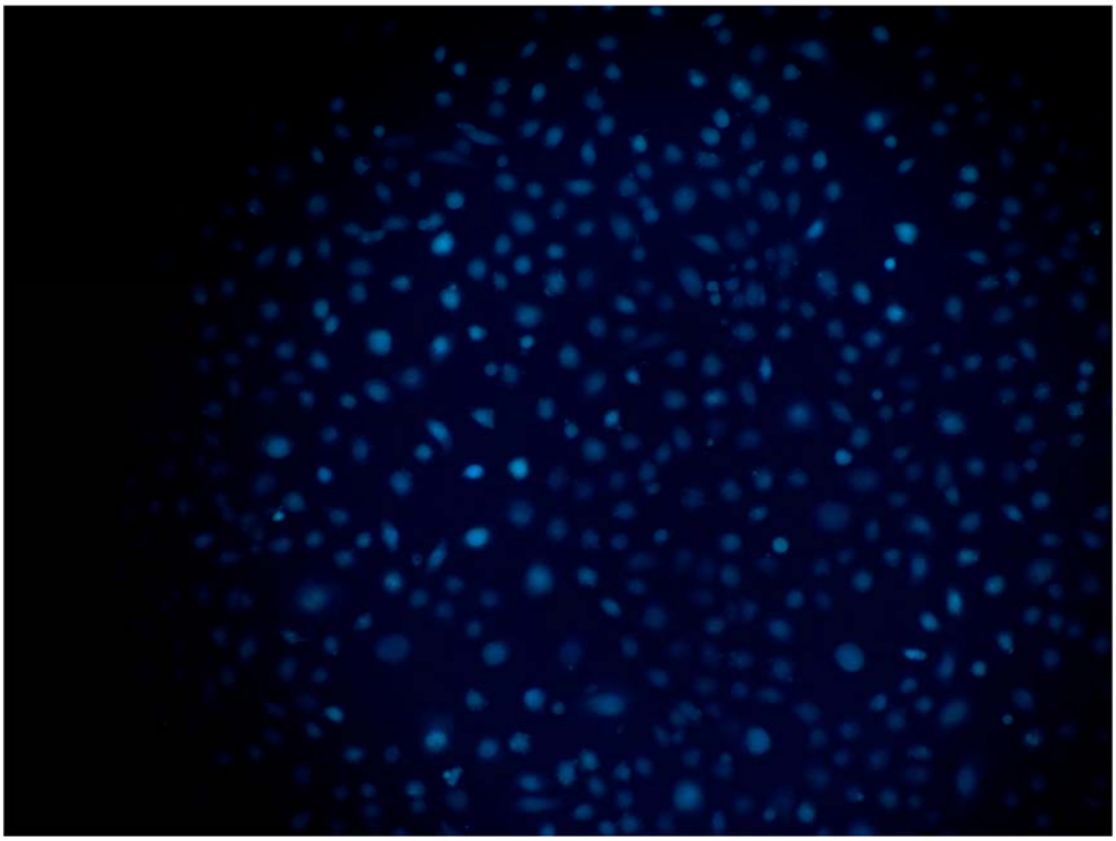

Figure S2. Video: Time-Dependent Fluorescence Intensity of Indo-1 Changes upon PbAc Solution Addition. Scale bar: 50 µm.

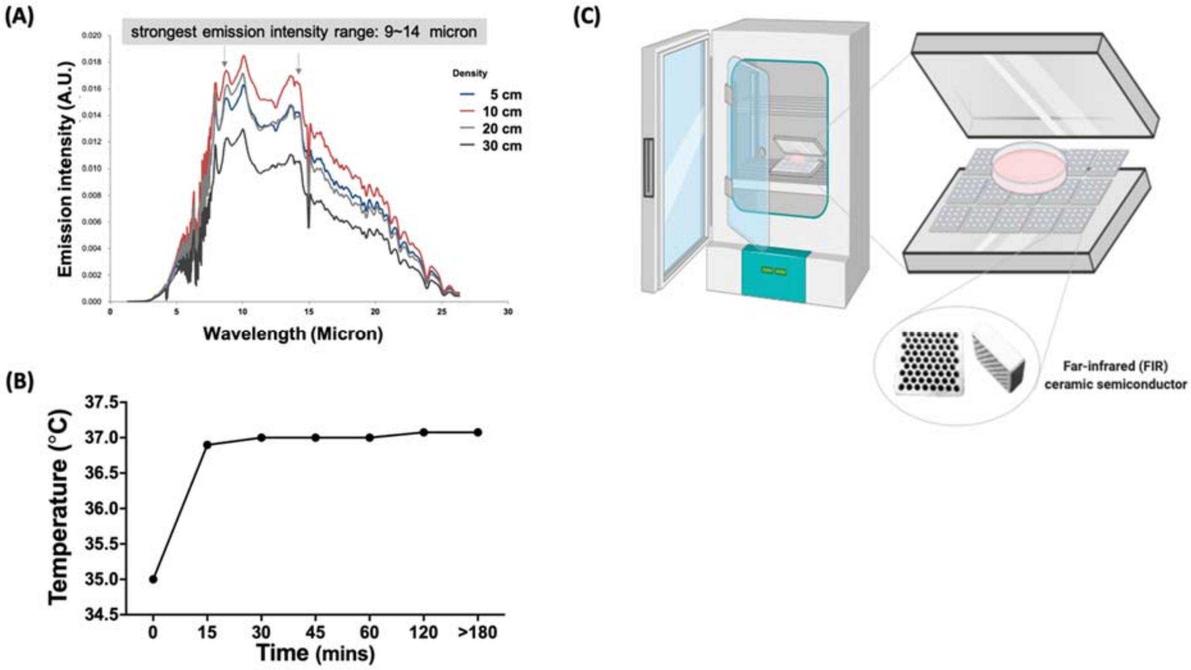

Figure S3. The wavelength and temperature of far-infrared (FIR) ceramic. (A) Far infrared range measurement by Fourier-transform infrared spectroscopy (FTIR) **(B)** Temperature of culture environment was measured directly by the thermometer **(C)** The schematic diagram of FIR device setting in cell culture.
